# Current Role of PET CT in Staging and Management of Penile Cancers

**DOI:** 10.3390/jcm13164879

**Published:** 2024-08-18

**Authors:** Cristian Mirvald, Radion Garaz, Ioanel Sinescu, Adrian Preda, Apostolos Labanaris, Ofer Yossepowitch, Igor Tsaur, Cristian Surcel

**Affiliations:** 1 Department of Urology, Fundeni Clinical Institute, 022328 Bucharest, Romaniaa.t.preda@gmail.com (A.P.);; 2 “Carol Davila” University of Medicine and Pharmacy, 050474 Bucharest, Romania; 3 Department of Urology, University Hospital Tübingen, 72076 Tübingen, Germany; 4 Department of Urology, Interbalkan Medical Center, 57001 Thessaloniki, Greece; 5 Department of Urology, Tel Aviv Sourasky Medical Center, Tel Aviv 64239, Israel

**Keywords:** penile cancer, PET CT, lymph nodes, sentinel node biopsy

## Abstract

Penile cancer (PeCa) is a rare urological malignancy characterized by significant geographical variations in both incidence and mortality rates. Due to its rarity and the consequent lack of randomized trials, current management is based on retrospective studies and small prospective trials. In addition, both the diagnostic pathways and treatment strategies exhibit substantial heterogeneity, differing significantly between less-developed and well-developed countries. The prognosis of PeCas is determined by the presence and extent of regional lymph node (LN) involvement. Therefore, the early detection and treatment of LN metastasis is paramount to ensure better outcomes. In recent decades, overall survival of PeCas has increased, mainly due to advancements in imaging techniques and risk stratification. We aim to provide an overview of the current role of PET CT imaging in the management of patients with PeCa.

## 1. Introduction

Penile cancer (PeCa) is a rare urological malignancy with significant geographical variations in incidence and mortality [1]. Squamous cell carcinoma (SCC) accounts for approximately 95% of PeCas; however, sarcoma, melanoma, and basal cell carcinoma have also been reported [1,2]. Due to its rarity, and the consequent lack of randomized trials, current therapies are based on retrospective studies and small prospective trials. In addition, both the diagnostic pathways and therapeutic management options are heterogeneous and vary significantly between poor and well-developed countries [1,3,4]. However, over the past decades, overall survival (OS) of PeCas has increased, mainly due to significant improvements in imaging and risk stratification [5]. 

The presence and extent of regional lymph node determines the prognosis of PeCas. Thus, the early detection and treatment of lymph node (LN) metastasis is paramount to ensur better outcomes [4]. Noninvasive N and M staging currently relies on ultrasonography, computed tomography (CT), and magnetic resonance imaging (MRI). These imaging techniques, however, are not sufficiently robust for detecting inguinal metastases, with 20–25% of cN0 patients harboring occult metastases [4,5]. Positron emission tomography (PET), combined with CT, may provide additional anatomic and metabolic information for staging and assessing treatment responses. It is currently used in the management of several malignancies, such as lung or head–neck cancers, which are mainly SCC, similar to PeCas [6,7]. PET imaging using ^18^F-fluorodeoxyglucose (FDG) relies on the increased cellular uptake of glucose and FDG, particularly in malignant cells and other tissues characterized by heightened glycolytic activity [8].

The role of PET imaging in penile cancers has not been adequately explored. Our aim is to assess the current role of PET/CT imaging in the management of patients with PeCa.

## 2. Materials and Methods

**Figure 1:**
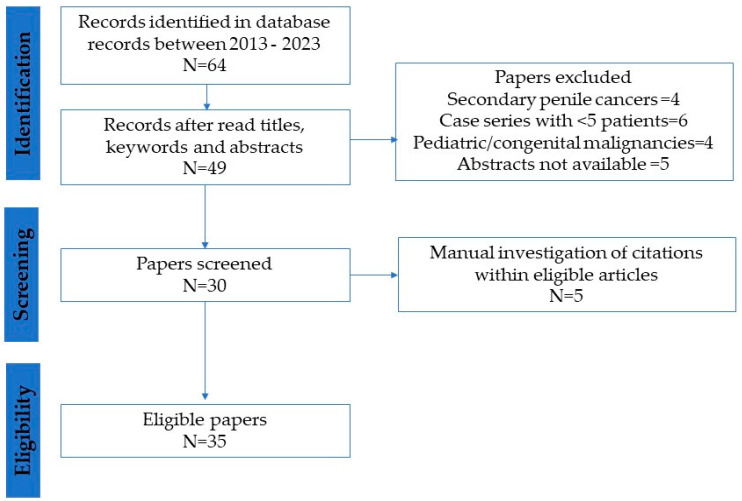
Flowchart of paper selection process.

An extensive, non-systematic literature search of the Scopus, PubMed/MEDLINE, and Web of Science^TM^ databases was conducted to identify recent manuscripts, published between 2013 and 2023, related to PET/CT imaging of primary PeCas. Key search words included “penile cancer”, “penile squamous cell carcinoma”, “PET CT”, “18 FDG”, “lymph nodes”, “clinically node negative”, “clinically node positive”, and “enlarged nodes”. There were no filters for language. Studies that evaluated pediatric/congenital malignancies or secondary penile cancers, consisted of case series with <5 patients, or were not exclusive to primary PeCas were excluded (N = 30). A manual investigation of the citations within the identified articles was also performed (N = 5). Systemic standards, including both the impact factor and citation frequency, were considered when citing other review studies. The flowchart outlining the study selection process for eligible papers is presented in Figure 1.

## 3. Results

### 3.1. Role of PET-CT for T Staging in Penile Cancer

Physical examination, including palpation, has traditionally been and remains the cornerstone for evaluating the extent of the primary tumor (T stage). In addition, imaging plays a crucial role when considering penile preservation surgery. For the detection of corpora cavernosa invasion, ultrasound exhibits a sensitivity of 97% and a specificity of 99%, whereas MRI shows a sensitivity of 74% and a specificity of 99% [9]. 

In a Brazilian study that included 53 patients, a higher pSUVmax was observed in more advanced tumors (pT1b and above) and in tumors with poorer differentiation compared to those with less aggressive lesions (*p* < 0.019). Several pathological features, such as dartos infiltration, lamina propria invasion, or perineural invasion, were associated with increased FDG uptake [10]. Although almost all PeCas present a certain degree of FDG uptake, small lesions can be missed due to limited spatial resolution and urine leakage, which can mask the tumor [11]. Due to these limitations, PET/CT cannot be recommended for initial T staging. 

In summary, when considering organ-sparing treatments for PeCa patients, an ultrasound with Doppler emerges as the preferred method for detecting corporal invasion. An MRI can also be considered as an alternative [12]. In surgically treated patients, an uptake appearing within the foreskin or shaft may be regarded as either a residual or recurrent tumor, as opposed to an artifact, which may be amenable for surgery [11]. In a small case series of 13 patients, Musi et al. demonstrated that local recurrences can be safely treated with salvage surgery, either by local excision or laser ablation, without compromising oncological control of the disease [13]. However, the data are insufficient to establish the role of PET/CT for assessing the primary tumor in initial and recurrent scenarios.

### 3.2. Role of PET-CT in the Management of Clinically Node-Negative PeCas

The presence and the extent of regional inguinal lymph node (ILN) metastases are regarded as the most important prognostic indicator for determining the long-term survival in men with invasive PeCa [4,5,12]. Furthermore, node-negative (cN0) patients still have a 13–16% chance of harboring occult metastases, whereas node-positive (cN+) patients have a 20–40% chance of being metastasis-free [14].

Research has shown that between 11% and 60% of lymph node metastasis are missed during initial screening, as micrometastases frequently occur and do not enlarge the node sufficiently to be detected on palpation [15].

Conventional cross-sectional imaging techniques, such as CT and MRI, often rely on size criteria (e.g., >8–10 mm) to diagnose metastatic spread to lymph nodes. However, using size criteria alone can result in a significant number of cancerous nodes being missed, while benign nodes may be falsely identified as positive [16]. Graafland et al. examined their experience with CT imaging to detect metastatic spread to inguinal and pelvic lymph nodes. In patients with a low risk for inguinal nodal involvement, an 8 mm cut-off in the short axis of the node provided the highest accuracy for predicting a positive node, with a sensitivity of 87% and specificity of 81% [16]. MRI can also stage ILNs. Although, MRI may not offer significant additional information over CT scans for ILN imaging, it can provide valuable insights when staging the primary tumor [16]. Lucchesi et al. found ILN involvement in 13 out of 15 cases (86.7%) using MRI, compared to physical examinations that identified only 7 of the 15 nodes (46.7%) [17].

Several studies assessed the use of FDG PET/CT to detect inguinal involvement in PeCa patients with non-palpable lymph nodes that were initially classified as node-negative (cN0). Salazar et al. used PET/CT imaging in a mixed cN0 and cN1 cohort of 53 patients. With an SUVmax cut-off of 6.5, they reported a sensitivity of 77% and a specificity of 78% when compared to histopathological results after inguinal lymph node dissection (ILND) [10]. A recent study investigated 18F-FDG PET/CT for ILN staging in cN0-only patients (n = 41) and reached a patient-based sensitivity and specificity of 80% and 68%, respectively, similar to the results of Salazar et al. [18]. Although the sensitivity of these new studies is higher than that reported by a meta-analysis of seven trials conducted by Sadeghi et al. in 2012 of only 57% per groin, none of these studies explicitly compared FDG PET/CT with other imaging modalities, such as ultrasound, which present with a similar accuracy to PET/CT in this clinical scenario [19]. 

Kroon and colleagues conducted fine-needle aspiration cytology (FNAC) prior to dynamic sentinel node biopsy (SNB) or ILND in 83 cN0 PeCa patients. They found that ultrasound with FNAC had both a sensitivity and specificity of 100% [20].

Jakobsen et al. evaluated the diagnostic accuracy of a sentinel node biopsy (SNB) combined with preoperative 18F-FDG PET/CT in a cohort of 61 cN0 PeCa patients. They reported a combined FDG PET/CT-SNB sensitivity of 94.4% (95% confidence interval [CI] 81–99%) per groin and a false-negative rate of 5.6% (95% CI 1–19%) per groin [21]. Despite promising results and the low rate of adverse events after an SNB, the false-negative rate of an SNB is still relatively high, suggesting that more trials are needed to demonstrate the clinical utility of this combined diagnostic approach. The incorporation of fluorescence imaging into the SNB procedure, utilizing the hybrid fluorescence and radioactive tracer indocyanine green (ICG)-99mTc-nanocolloid, has been demonstrated to improve the intraoperative localization of sentinel nodes in PeCas [22]. As observed in other cancers, high 18F-FDG uptake in the primary tumor frequently leads to decreased uptake in the regional nodes, suggesting that PET imaging assessment in node-negative patients should be conducted post-primary tumor removal to enhance the likelihood of LN detection. However, this approach is hindered by elevated costs and an increased risk of false positives rates due to postoperative inflammation [23].

In conclusion, in cN0, although FDG PET/CT reveals a significant negative predictive value (NPV), it cannot replace invasive nodal staging due to its inadequate sensitivity (particularly regarding micrometastases) and specificity (i.e., in discriminating between (post)inflammatory and metastatic lymph nodes).

### 3.3. Role of PET-CT in the Management of Clinically Node-Positive PeCas

**Figure 2:**
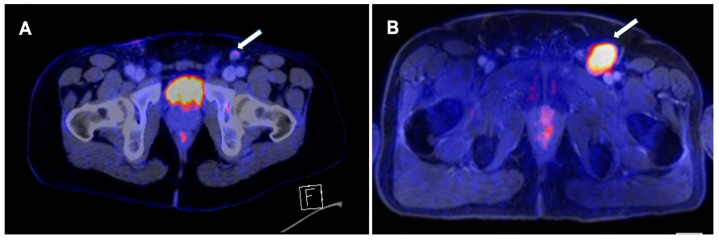
(**A**). A 37-year-old man with stage T2 penile squamous cell carcinoma. Imaging with 18FDG PET/CT shows one 12 mm, SUV_mean_ 2 suspect left inguinal lymph node (Arrow). Histology revealed a metastasis of PeCa without extracapsular extension. (**B**). A 61-year-old man with stage T1 penile squamous cell carcinoma. Imaging with 18FDG-PET/MR shows one 40 × 26 mm SUVmax 7 positive left inguinal lymph node. Histology revealed a metastasis of PeCa without extracapsular extension.

Palpable lymphadenopathy warrants prompt evaluation, as the risk of metastasis is high (45–80%) [4]. Although the early treatment of lymph node involvement has been shown to positively impact survival, the presence of clinically nodal disease at diagnosis does not warrant an immediate ILND since approximately 50% of patients present with inflammatory swelling instead of metastatic spread [4,24]. PET/CT demonstrates a strong diagnostic performance in this context, as shown in Figure 2.

In a recently published meta-analysis that evaluated 12 studies (479 patients), the pooled sensitivity of 18F-FDG PET/CT was 0.87 (95% confidence interval [CI], 0.79–0.92), and the pooled specificity was 0.88 (95% CI, 0.79–0.93) [25]. These results are better than those reported in the meta-analysis conducted by Sadeghi et al. in 2012 [19]. These differences could be related to the use of SUV cut-off points, since this methodology was not used in the previous analysis, which was based exclusively on qualitative analyses [10].

Salazar et al. demonstrated that the semiquantitative PET/CT parameters (pSUVmax and nSUVmax) may have a prognostic value. Their cohort reported that a pSUVmax of 16.6 was the best predictor for OS (*p* = 0.0001), followed by a nSUVmax of 6.5 (*p* = 0.019) [10]. Although these PET parameters are already used in other squamous cancers, these results should be carefully considered, with further evaluations being necessary to consolidate these findings in this clinical setting [26,27].

In cases where an inguinal biopsy is not always feasible due to patient refusal or the risk of damaging the femoral vessels, FDG PET/CT can be a useful tool in assessing the nature of a suspicious lesion. Zhang and colleagues reported that in a cohort of 48 cN1 patients, FDG PET/CT detected more malignant diseases than either CT or MRI in 33% of cases and treatment was changed after PET/CT results in 57% of patients [28].

Primary surgery is not recommended in the case of fixed, bilateral, or large nodal illness due to the poor prognosis [4]. A multimodal approach, including neoadjuvant chemotherapy followed by consolidative surgery, is recommended in cases with complete or partial responses. In this setting, the impact of neoadjuvant treatments may be assessed by PET/CT. Ottenhof et al. reported on the safety and efficacy of chemoradiotherapy (CRT) as the primary treatment for nonmetastatic patients with large/inoperable primary tumors and large palpable nodes in a prospective, single-center, single-arm study [29]. In their cohort of 33 patients, the impact of CRT was assessed using 18F-FDG PET/CT. The authors reported that a response was achieved in 73% (n = 24) of patients, and within this group, 13 (39%) patients achieved a complete response. Despite the authors reporting only short-term outcomes (2-year OS of 46%), these results are promising, considering the reported 5-year survival rate of less than 20% for this cohort with a poor prognosis [4,24]. Nevertheless, the presence of elevated FDG uptake and LN metastases in most primary penile tumors indicates that FDG PET/CT is a valuable tool for staging PeCas [5].

Copper is a transitional metal that plays a role in the signal transduction pathways regulating cancer cell proliferation and tumor growth [30]. Radioactive copper-64 chloride (64CuCl2) is a useful radiotracer for cancer imaging with PET due to the increased cellular uptake of cooper mediated by the human copper transporter 1, which is expressed on the cancer cell membrane. To increase the diagnostic accuracy of PET/CT, Mascia et al. reported the results of 64(II)dichloride (64Cu(II)Cl2) as a new PET radiotracer for urological malignancies (prostate cancer, bladder cancer, penile cancer, and kidney cancer) in a phase 2 clinical trial [26]. In this study, the authors had a small sample size of only 6 PeCa patients. The detection rate of nodal disease in PeCas was 83.3% (5/6) with an area under the curve of 0.775 (SUVmax 3.9). Although 64Cu(II)Cl2 is an effective and well-tolerated radiotracer in patients affected by prostate, bladder, and penile cancer, more trials are needed to confirm its utility in the management of node-positive PeCa patients. 

Many cancer-associated fibroblasts differ from normal fibroblasts due to their relatively specific expression of fibroblast activation protein (FAP) inhibitors [31,32]. Consequently, FAP-specific inhibitors were initially developed as anticancer drugs and subsequently advanced into tumor-targeting therapies. A biodistribution and initial dosimetry study of a 68Ga-FAP inhibitor (FAPI) PET/CT with two DOTA-containing ligands suggested that these tracers could expand and enhance the diagnostic capabilities currently covered by 18F-FDG [33]. Several epidemiologically significant tumor types, particularly breast, esophageal, lung, pancreatic, head–neck, and colorectal cancer, exhibit remarkably high uptake in 68Ga-FAPI PET/CT [32]. Moreover, in a pilot study aimed at preoperative assistance, 11 histologically confirmed PeCa patients underwent staging with [68Ga]Ga-FAPI-46 PET/CT before surgery. The histologically confirmed lymph node regions showed significantly elevated FAPI uptakes (SUVmax 17.9, range 16.4–23.5), with no instances of false-positive FAPI uptake. This potentially enables the detection of occult LN metastases [27]. 

### 3.4. Role of PET CT in the Management of Pelvic Lymph Nodes and Distant Metastasis

Pelvic LN (PLN) metastases following ILN spread are associated with a worse prognosis, with a 5-year disease-specific survival rate of 17% compared to 62% in cases without PLN metastases [6]. Adjuvant radiotherapy and chemotherapy have been shown to improve outcomes following pelvic lymph node dissection for nodal metastases [34]. At the time of diagnosis of PeCa, only 1% to 10% of cases present with distant metastases [35].

The literature regarding the impact of PET CT on the detection and management of pelvic LNs and metastasis is scarce and mainly comprises case presentations or retrospective series. In the aforementioned study by Zhang and collaborators, the sensitivity and specificity of FDG PET/CT were 85% and 86%, respectively, for all metastatic sites, including the lymph nodes, lung, liver, bone, and brain, using histopathology or follow-up imaging as a reference [28]. Ottenhof et al. reported that in their cohort of 61 high-risk PeCa patients, PET/CT demonstrated a sensitivity of 85% for pelvic LN staging (a specificity of 75%, NPV of 90%, and PPV of 65%) and a PPV of 93% for the detection of distant metastases, suggesting that FDG PET/CT imaging should be used in the initial diagnosis of PeCas to avoid the futile treatment of patients with distant metastases [6]. 

### 3.5. Role of PET CT in Follow-Up after Treatment

**Figure 3:**
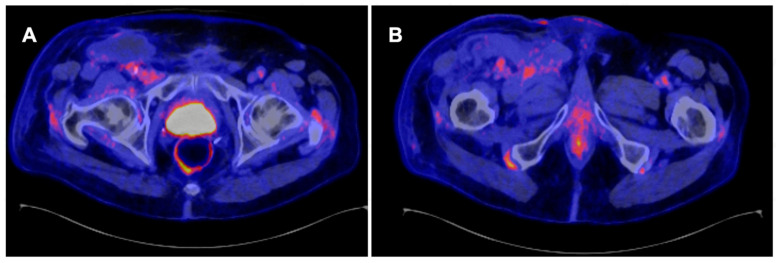
A 71-year-old man with stage pT1 pN1 penile squamous cell carcinoma shows no evidence of positive pelvic lymph nodes (**A**) or inguinal lymph nodes (**B**) during follow-up.

Local or nodal recurrences usually occur within 2–3 years of primary treatment. This suggests a rigorous follow-up schedule for the first two years, followed by less intense follow-up for at least five years [4,11]. Current guidelines recommend that PET/CT may be an alternative to CT or MRI for imaging follow-up after definitive treatment [4,36], as shown in Figure 3. In comparison to other imaging tests, PET/CT is usually more costly, and its availability may be a limiting factor in some countries [37]. Another drawback of PET/CT for systemic staging is that it is frequently conducted without intravenous contrast, which makes measuring the extent of visceral metastases difficult [24].

### 3.6. Implications for Further Research

Given the heterogeneous nature of the included studies, the preponderance of noncomparative data, and the small sample sizes, it is challenging to provide conclusive quantitative results for the research questions. Limited data are available evaluating the role and efficacy of PET/CT in the diagnosis and management of PeCas. This underscores the need for high-quality, comparative randomized studies, which is expected to remain a challenge in this rare disease.

The ongoing International Penile Advanced Cancer Trial (InPACT, NCT02305654) is a phase 3 trial, with a Bayesian design, that is incorporating two sequential randomizations to address two main questions. The aim is to recruit 200 patients with inguinal and/or pelvic metastases. The first randomization will evaluate the role of neoadjuvant therapies before ILND. The second randomization concerns the role of prophylactic pelvic LND following standard surgical treatment with therapeutic ILND in patients with high pathological risk factors after receiving chemotherapy [38]. In these scenarios, there will be a great opportunity to include PET/CT as a radiological tool for detecting the radiological response. Bandini and colleagues conducted a multicenter study involving 334 cN+ PeCa patients, of whom 48 patients (14.4%) underwent PET/CT [39]. Their findings revealed that 18FDG PET/CT can be used to stratify patients during the preoperative evaluation. PeCa patients with cN3 or cN2 disease, as detected by PET/CT, exhibited inguinal and pelvic nodal activity and had a higher risk of 24-month overall mortality (>50%). In these patients, the use of neoadjuvant chemotherapy appears to be effective and can be administrated to improve survival.

Moreover, additional studies are needed to confirm the safety and effectiveness of 64Cu(II)Cl2 and 68Ga-FAPI radiotracers for the management of node-positive PeCa patients.

## 4. Conclusions

Imaging with 18F-FDG PET plays a pivotal role in the management of clinically node-positive penile cancer patients and may serve as an independent prognostic factor for survival in this population. In addition, PET/CT can assist in surgical planning and evaluating chemotherapy responses. Integrating 18F-FDG PET/CT into future staging algorithms has the potential to guide more precise and stage-appropriate therapeutic strategies. 

## References

[1] Fu L., Tian T., Yao K., Chen X.-F., Luo G., Gao Y., Lin Y.-F., Wang B., Sun Y., Zheng W. (2022). Global Pattern and Trends in Penile Cancer Incidence: Population-Based Study. JMIR Public Health Surveill..

[2] Moch H., Cubilla A.L., Humphrey P.A., Reuter V.E., Ulbright T.M. (2016). The 2016 WHO Classification of Tumours of the Urinary System and Male Genital Organs-Part A: Renal, Penile, and Testicular Tumours. Eur. Urol..

[3] Hogan D., Norton S.M., Patterson K., Murphy A., O‘Neill B., Daly P., Cullen I.M. (2024). Phallus preservation and reconstruction: 5-year outcomes of national penile cancer centralisation in the Republic of Ireland. Surgeon.

[4] Brouwer O.R., Albersen M., Parnham A., Protzel C., Pettaway C.A., Ayres B., Antunes-Lopes T., Barreto L., Campi R., Crook J. (2023). European Association of Urology-American Society of Clinical Oncology Collaborative Guideline on Penile Cancer: 2023 Update. Eur. Urol..

[5] Ottenhof S.R., Leone A.R., Horenblas S., Spiess P.E., Vegt E. (2017). Advancements in staging and imaging for penile cancer. Curr. Opin. Urol..

[6] Ottenhof S.R., Djajadiningrat R.S., Versleijen M.W.J., Donswijk M.L., van der Noort V., Brouwer O.R., Graafland N.M., Vegt E., Horenblas S. (2022). F-18 Fluorodeoxyglucose Positron Emission Tomography with Computed Tomography Has High Diagnostic Value for Pelvic and Distant Staging in Patients with High-risk Penile Carcinoma. Eur. Urol. Focus.

[7] Reske S.N., Kotzerke J. (2001). FDG-PET for clinical use. Results of the 3rd German Interdisciplinary Consensus Conference, “Onko-PET III”, 21 July and 19 September 2000. Eur. J. Nucl. Med..

[8] Fletcher J.W., Djulbegovic B., Soares H.P., Siegel B.A., Lowe V.J., Lyman G.H., Coleman R.E., Wahl R., Paschold J.C., Avril N. (2008). Recommendations on the use of ^18^F-FDG PET in oncology. J. Nucl. Med..

[9] Bozzini G., Provenzano M., Otero J.R., Margreiter M., Cruz E.G., Osmolorskij B., Verze P., Pavan N., Sanguedolce F., Buffi N. (2016). Role of Penile Doppler US in the Preoperative Assessment of Penile Squamous Cell Carcinoma Patients: Results From a Large Prospective Multicenter European Study. Urology.

[10] Salazar A., Júnior E.P., Salles P.G.O., Silva-Filho R., Reis E.A., Mamede M. (2019). 18F-FDG PET/CT as a prognostic factor in penile cancer. Eur. J. Nucl. Med..

[11] Ottenhof S.R., Vegt E. (2017). The role of PET/CT imaging in penile cancer. Transl. Androl. Urol..

[12] De Vries H.M., Brouwer O.R., Heijmink S., Horenblas S., Vegt E. (2019). Recent developments in penile cancer imaging. Curr. Opin. Urol..

[13] Musi G., Molinari F., Mistretta F.A., Piccinelli M.L., Guzzo S., Tozzi M., Lievore E., Blezien O., Fontana M., Cioffi A. (2023). Penile-Sparing Surgery for Tumour Recurrence after Previous Glansectomy/Partial Penectomy: Treatment Feasibility and Oncological Outcomes. Cancers.

[14] Hughes B., Leijte J., Shabbir M., Watkin N., Horenblas S. (2009). Non-invasive and minimally invasive staging of regional lymph nodes in penile cancer. World J. Urol..

[15] Wood H.M., Angermeier K.W. (2010). Anatomic considerations of the penis, lymphatic drainage, and biopsy of the sentinel node. Urol. Clin. N. Am..

[16] Bloom J.B., Stern M., Patel N.H., Zhang M., Phillips J.L. (2018). Detection of lymph node metastases in penile cancer. Transl. Androl. Urol..

[17] Lucchesi F.R., Reis R.B., Faria E.F., Machado R.D., Rossini R.R., Borregales L.D., Silva G.E.B., Muglia V.F. (2017). Incremental value of MRI for preoperative penile cancer staging. J. Magn. Reson. Imaging.

[18] Drager D.L., Heuschkel M., Protzel C., Erbersdobler A., Krause B.J., Hakenberg O.W., Schwarzenbock S.M. (2018). [18F]FDG PET/CT for assessing inguinal lymph nodes in patients with penile cancer-correlation with histopathology after inguinal lymphadenectomy. Nuklearmedizin.

[19] Sadeghi R., Gholami H., Zakavi S.R., Kakhki V.R., Horenblas S. (2012). Accuracy of 18F-FDG PET/CT for diagnosing inguinal lymph node involvement in penile squamous cell carcinoma: Systematic review and meta-analysis of the literature. Clin. Nucl. Med..

[20] Kroon B.K., Horenblas S., Deurloo E.E., Nieweg O.E., Teertstra H.J. (2005). Ultrasonography-guided fine-needle aspiration cytology before sentinel node biopsy in patients with penile carcinoma. BJU Int..

[21] Jakobsen J.K., Alslev L., Ipsen P., Costa J.C., Krarup K.P., Sommer P., Nerstrøm H., Toft B.G., Høyer S., Bouchelouche K. (2016). DaPeCa-3: Promising results of sentinel node biopsy combined with ^18^F-fluorodeoxyglucose positron emission tomography/computed tomography in clinically lymph node-negative patients with penile cancer—A national study from Denmark. BJU Int..

[22] KleinJan G.H., van Werkhoven E., Berg N.S.v.D., Karakullukcu M.B., Zijlmans H.J.M.A.A., van der Hage J.A., van de Wiel B.A., Buckle T., Klop W.M.C., Horenblas S. (2018). The best of both worlds: A hybrid approach for optimal pre- and intraoperative identification of sentinel lymph nodes. Eur. J. Nucl. Med..

[23] van Westreenen H., Westerterp M., Bossuyt P., Pruim J., Sloof G., van Lanschot J., Groen H., Plukker J. (2004). Systematic review of the staging performance of^18^F-fluorodeoxyglucose positron emission tomography in esophageal cancer. J. Clin. Oncol..

[24] Peyraud F., Allenet C., Gross-Goupil M., Domblides C., Lefort F., Daste A., Yacoub M., Haaser T., Ferretti L., Robert G. (2020). Current management and future perspectives of penile cancer: An updated review. Cancer Treat. Rev..

[25] Lee S.W., Kim S.-J. (2022). Diagnostic Performance of 18F-FDG PET/CT for Lymph Node Staging in Penile Cancer. Clin. Nucl. Med..

[26] Chu K.P., Murphy J.D., La T.H., Krakow T.E., Iagaru A., Graves E.E., Hsu A., Maxim P.G., Loo B., Chang D.T. (2012). Prognostic value of metabolic tumor volume and velocity in predicting head-and-neck cancer outcomes. Int. J. Radiat. Oncol..

[27] Yoo Ie R., Chung S.K., Park H.L., Choi W.H., Kim Y.K., Lee K.Y., Wang Y.P. (2014). Prognostic value of SUVmax and metabolic tumor volume on 18F-FDG PET/CT in early stage non-small cell lung cancer patients without LN metastasis. Biomed. Mater. Eng..

[28] Zhang S., Li W., Liang F. (2016). Clinical value of fluorine-18 2-fluoro-2-deoxy-D-glucose positron emission tomography/computed tomography in penile cancer. Oncotarget.

[29] Ottenhof S.R., de Vries H.M., Doodeman B., Vrijenhoek G.L., van der Noort V., Donswijk M.L., de Feijter J.M., Schaake E.E., Horenblas S., Brouwer O.R. (2023). A Prospective Study of Chemoradiotherapy as Primary Treatment in Patients With Locoregionally Advanced Penile Carcinoma. Int. J. Radiat. Oncol. Biol. Phys..

[30] Peng F. (2022). Recent Advances in Cancer Imaging with 64CuCl2 PET/CT. Nucl. Med. Mol. Imaging.

[31] Loktev A., Lindner T., Mier W., Debus J., Altmann A., Jäger D., Giesel F., Kratochwil C., Barthe P., Roumestand C. (2018). A Tumor-Imaging Method Targeting Cancer-Associated Fibroblasts. J. Nucl. Med..

[32] Kratochwil C., Flechsig P., Lindner T., Abderrahim L., Altmann A., Mier W., Adeberg S., Rathke H., Röhrich M., Winter H. (2019). ^68^Ga-FAPI PET/CT: Tracer Uptake in 28 Different Kinds of Cancer. J. Nucl. Med..

[33] Giesel F.L., Kratochwil C., Lindner T., Marschalek M.M., Loktev A., Lehnert W., Debus J., Jäger D., Flechsig P., Altmann A. (2019). ^68^Ga-FAPI PET/CT: Biodistribution and Preliminary Dosimetry Estimate of 2 DOTA-Containing FAP-Targeting Agents in Patients with Various Cancers. J. Nucl. Med..

[34] Chen W.-K., Wu Z.-G. (2020). Adding radiotherapy based on chemotherapy can improve cancer-specific survival in N3 penile cancer: A SEER-based study. Transl. Androl. Urol..

[35] Chahoud J., Kohli M., Spiess P.E. (2021). Management of Advanced Penile Cancer. Mayo Clin. Proc..

[36] Clark P.E., Spiess P.E., Agarwal N., Biagioli M.C., Eisenberger M.A., Greenberg R.E., Herr H.W., Inman B.A., Kuban D.A., Kuzel T.M. (2013). Penile cancer: Clinical Practice Guidelines in Oncology. J. Natl. Compr. Canc. Netw..

[37] Campbell R.A., Slopnick E.A., Ferry E.K., Zhu H., Kim S.P., Abouassaly R. (2017). Disparity between pre-existing management of penile cancer and NCCN guidelines. Urol. Oncol. Semin. Orig. Investig..

[38] Pettaway C.A., Nicholson S., Spiess P.E., Pagliaro L.C., Watkin N., Barber J., Carducci M.A., Trabulsi E.J., Crook J.M., Rosen M.A. (2022). The international penile advanced cancer trial (InPACT): The first phase III trial for squamous carcinoma of the penis with regional lymph node metastases. J. Clin. Oncol..

[39] Bandini M., Albersen M., Chipollini J., Pederzoli F., Zhu Y., Ye D., Ornellas A.A., Watkin N., Ager M., Hakenberg O.W. (2020). Optimising the selection of candidates for neoadjuvant chemotherapy amongst patients with node-positive penile squamous cell carcinoma. BJU Int..

